# Incidental rib hotspots on 99mTc-pyrophosphate scintigraphy in a patient with transthyretin cardiac amyloidosis: cough-induced fractures unveiled

**DOI:** 10.1093/ehjcr/ytaf472

**Published:** 2025-09-18

**Authors:** Satoshi Kurisu, Hitoshi Fujiwara

**Affiliations:** Department of Cardiology, NHO Hiroshimanishi Medical Center, 4-1-1, Kuba, Otake-shi, Hiroshima 739-0696, Japan; Department of Cardiology, NHO Hiroshimanishi Medical Center, 4-1-1, Kuba, Otake-shi, Hiroshima 739-0696, Japan

## Case description

**Figure 1:**
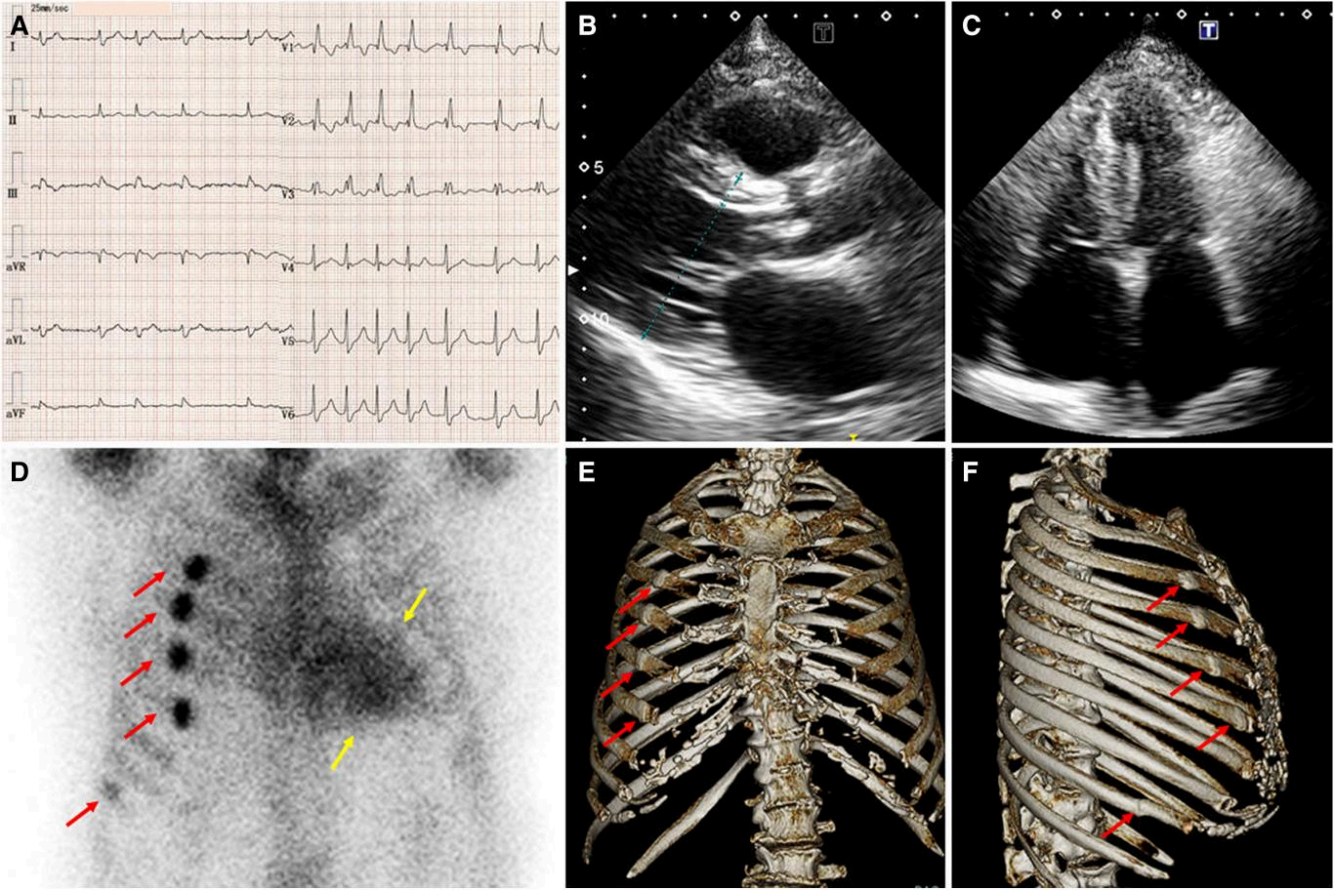
Electrocardiography showed atrial fibrillation, low-voltage complexes, and right bundle branch block (*A*). Transthoracic echocardiography revealed increased left ventricular wall thickness of 12 mm (*B*) and increased left atrial volume index of 48 mL/m^2^ (*C*). Technetium-99 m pyrophosphate scintigraphy demonstrated Perugini Grade 3 myocardial uptake (*D*, yellow arrows). Unexpectedly, five focal extracardiac hotspots were observed: four along the anterolateral right third to sixth ribs and one at the lateral right ninth rib (*D*, red arrows). Chest computed tomography demonstrated focal sclerotic changes at the third to sixth rib sites on frontal and lateral images (*E* and *F*, arrows), consistent with healed rib fractures. At the ninth rib site, a fracture line was clearly visualized on lateral imaging (*F*, arrow), confirming an additional rib fracture.

An 88-year-old woman was hospitalized for an asthma exacerbation. She had lumbar canal stenosis and bilateral carpal tunnel syndrome but no family history of heart disease. After a severe coughing episode, she was treated with intravenous corticosteroids. Two months later, she was readmitted for heart failure. Electrocardiography showed atrial fibrillation, low-voltage complexes, and right bundle branch block (*Figure 1A*). Transthoracic echocardiography revealed increased left ventricular wall thickness of 12 mm (*Figure 1B*), preserved ejection fraction of 58%, and increased left atrial volume index of 48 mL/m^2^ (*Figure 1C*), raising suspicion for cardiac amyloidosis. Technetium-99m pyrophosphate (99mTc-PYP) scintigraphy demonstrated Perugini Grade 3 myocardial uptake (*Figure 1D*, yellow arrows). Serum and urine immunofixation were negative for monoclonal proteins, and transthyretin genotyping was not performed. The diagnosis of transthyretin cardiac amyloidosis was made,^1^ and treatment with azosemide, dapagliflozin, and apixaban was initiated.

Unexpectedly, five focal extracardiac hotspots were observed: four along the anterolateral right third to sixth ribs and one at the lateral right ninth rib (*Figure 1D*, red arrows). Because of these hotspots, the heart-to-contralateral ratio could not be reliably measured. Chest computed tomography demonstrated focal sclerotic changes at the third to sixth rib sites on frontal and lateral images (*Figure 1E* and *F*, arrows), consistent with healed rib fractures. At the ninth rib site, a fracture line was clearly visualized on lateral imaging (*Figure 1F*, arrow), confirming an additional rib fracture. Although the exact timing of injury could not be confirmed, the fractures were suspected to be related to the recent coughing episode.

Cough-induced rib fractures are most commonly observed in postmenopausal women and are associated with risk factors such as asthma, osteoporosis, and corticosteroid use.^2,3^ This case underscores the importance of assessing extracardiac findings on 99mTc-PYP scintigraphy, as they may provide clinically meaningful insights beyond myocardial assessment.

## Data Availability

The data that support the findings of this study are available from the corresponding author upon reasonable request.
